# Abdominal obesity phenotypes are associated with the risk of developing non-alcoholic fatty liver disease: insights from the general population

**DOI:** 10.1186/s12876-022-02393-9

**Published:** 2022-06-25

**Authors:** Maobin Kuang, Song Lu, Qiyang Xie, Nan Peng, Shiming He, Changhui Yu, Jiajun Qiu, Guotai Sheng, Yang Zou

**Affiliations:** 1 Jiangxi Cardiovascular Research Institute, Jiangxi Provincial People’s Hospital, Nanchang, 330006 China; 2 Medical College of Nanchang University, Nanchang, 330006 China; 3 Cardiology Department, Jiangxi Provincial People’s Hospital, Nanchang, 330006 China

**Keywords:** Abdominal obesity, Metabolic syndrome, Abdominal obesity phenotypes, Metabolic health obesity, Non-alcoholic fatty liver disease

## Abstract

**Background:**

The diversity of obesity-related metabolic characteristics generates different obesity phenotypes and corresponding metabolic diseases. This study aims to explore the correlation of different abdominal obesity phenotypes with non-alcoholic fatty liver disease (NAFLD).

**Methods:**

The current study included 14,251 subjects, 7411 males and 6840 females. Abdominal obesity was defined as waist circumference ≥ 85 cm in males and ≥ 80 cm in females; according to the diagnostic criteria for metabolic syndrome recommended by the National Cholesterol Education Program Adult Treatment Panel III, having more than one metabolic abnormality (except waist circumference criteria) was defined as metabolically unhealthy. All subjects were divided into 4 abdominal obesity phenotypes based on the presence ( +) or absence (− ) of metabolically healthy/unhealthy (MH) and abdominal obesity (AO) at baseline: metabolically healthy + non-abdominal obesity (MH^−^AO^−^); metabolically healthy + abdominal obesity (MH^−^AO^+^); metabolically unhealthy + non-abdominal obesity (MH^+^AO^−^); metabolically unhealthy + abdominal obesity (MH^+^AO^+^). The relationship between each phenotype and NAFLD was analyzed using multivariate logistic regression.

**Results:**

A total of 2507 (17.59%) subjects in this study were diagnosed with NAFLD. The prevalence rates of NAFLD in female subjects with MH^−^AO^−^, MH^−^AO^+^, MH^+^AO^−^, and MH^+^AO^+^ phenotypes were 1.73%, 24.42%, 7.60%, and 59.35%, respectively. Among male subjects with MH^−^AO^−^, MH^−^AO^+^, MH^+^AO^−^, and MH^+^AO^+^ phenotypes, the prevalence rates were 9.93%, 50.54%, 25.49%, and 73.22%, respectively. After fully adjusting for confounding factors, with the MH^−^AO^−^ phenotype as the reference phenotype, male MH^−^AO^+^ and MH^+^AO^+^ phenotypes increased the risk of NAFLD by 42% and 47%, respectively (MH^−^AO^+^: OR 1.42, 95%CI 1.13,1.78; MH^+^AO^+^: OR 1.47, 95%CI 1.08,2.01); the corresponding risks of MH^−^AO^+^ and MH^+^AO^+^ in females increased by 113% and 134%, respectively (MH^−^AO^+^: OR 2.13, 95%CI 1.47,3.09; MH^+^AO^+^: OR 2.34, 95%CI 1.32,4.17); by contrast, there was no significant increase in the risk of NAFLD in the MH^+^AO^−^ phenotype in both sexes.

**Conclusions:**

This first report on the relationship of abdominal obesity phenotypes with NAFLD showed that both MH^−^AO^+^ and MH^+^AO^+^ phenotypes were associated with a higher risk of NAFLD, especially in the female population. These data provided a new reference for the screening and prevention of NAFLD.

**Supplementary Information:**

The online version contains supplementary material available at 10.1186/s12876-022-02393-9.

## Background

The liver is the most common site of visceral fat deposition. In recent years, with the epidemic of obesity and the drastic changes in diet and lifestyle, an increasing number of people around the world suffer from non-alcoholic fatty liver disease (NAFLD) [1]. Recent epidemiological surveys showed that the prevalence of NAFLD has reached 25.24% globally and 27.37% in Asia, and the overall prevalence of NAFLD is projected to reach 33.5% globally by 2030 [2, 3]. NAFLD doesn't just stop at hepatic steatosis; without interventions, NAFLD will gradually develop into liver fibrosis and even liver cancer [4, 5]. In addition, NAFLD can also cause a series of complications such as diabetes, cardiovascular disease, and digestive tract malignancies [6, 7]. These intrahepatic and extrahepatic lesions will affect the quality of life and survival time of NAFLD patients, and bring a heavy burden to the family and the world's medical and health systems [8]. Therefore, early identification of high-risk groups at risk for NAFLD is essential.

Obesity and metabolic disorders are typical clinical features and important risk factors of NAFLD patients, and insulin resistance (IR) is the main pathophysiological mechanism leading to NAFLD [9, 10]. However, not all obese people were at high risk for NAFLD; in recent years, several studies have found that obese people with healthy metabolic profiles were not at increased risk of cardiovascular disease and all-cause mortality, and their studies suggested that this particular metabolically healthy obesity (MHO) phenotype may be a good physical condition [11–13]. However, in further studies, more researchers found that the MHO phenotype was not a static obesity phenotype, with the extension of follow-up time, most patients with MHO phenotype would lose the state of metabolic health [14, 15]; this phenomenon was more common in patients with the abdominal obesity phenotype with a high waist circumference (WC), and visceral fat deposition appeared to contribute to this change [16–18]. WC is known to reflect central obesity, has better diagnostic performance than BMI in assessing visceral fat deposition and IR, and has also been shown to be a stronger anthropometric predictor of fatty liver [19, 20]. Therefore, we speculated that using abdominal obesity combined with metabolic profiles might be a more rational way to assess NAFLD risk, while also providing greater insight into the nature of different obesity phenotypes. However, the relationship between abdominal obesity phenotypes and NAFLD is currently unclear. The purpose of this study was to investigate the association of various abdominal obesity phenotypes with the NAFLD risk.

## Methods

### Study design and population

The current study was a secondary analysis based on data from the NAGALA cohort and adopted a cross-sectional design to explore the correlation of abdominal obesity phenotypes with NAFLD risk. The subjects' data in the current study were uploaded to the Dryad database by Hamaguchi et al. [21]. Based on the terms of service of the Dryad database, these subjects' data can be fully utilized under different scientific hypotheses. The detailed study design and procedures of the NAGALA cohort have been described elsewhere [22]. In short, NAGALA is an ongoing longitudinal cohort study based on the general population physical examination program launched by Murakami Memorial Hospital in 1994 to identify chronic diseases and their important risk factors that endanger the health of the general population. We extracted data from 14,251 examiners recruited by the NAGALA cohort between 1994 and 2015. According to the purpose and design of this study, we only included subjects who did not have the following characteristics: (1) absence of covariates; (2) diagnosed or self-reported viral/alcoholic hepatitis or diabetes at baseline; (3) taking medication or having impaired fasting glucose at baseline; (4) overdose alcohol consumption (Alcohol consumption ≥ 140 g/w for female or ≥ 210 g/w for male) [23]. Since Murakami Memorial Hospital has approved the ethical review of the previous cohort study [22], all subjects have signed informed consent forms, and their personal information has been replaced with a check code, therefore, this study does not need to apply for ethical approval and obtain informed consent repeatedly. All steps of this study are in full compliance with the Helsinki Declaration [24].

### Data collection and measurement

As previously described [22], trained medical examiners used a standard self-administered questionnaire to collect basic information about the subject's height, gender, blood pressure, WC, age, weight, and various lifestyle habits (exercise habits, smoking/drinking status).

Venous blood samples of the subjects were taken early the next morning after a night fast for at least 8 h and using an automatic analyzer analyzed for biochemical parameters including total cholesterol (TC), alanine aminotransferase (ALT), high-density lipoprotein cholesterol (HDL-C), gamma-glutamyl transferase (GGT), triglycerides (TG), hemoglobin A1c (HbA1c), fasting plasma glucose (FPG) and aspartate aminotransferase (AST).

### Variable definition

To define metabolic unhealthy, we referred to the National Cholesterol Education Program Adult Treatment Panel III diagnostic criteria for metabolic syndrome [25]: (1) FPG ≥ 5.6 mmol/L; (2) HDL-C < 1.29 mmol/L in females or < 1.03 mmol/L in males; (3) TG ≥ 1.7 mmol/ L; and (4) diastolic blood pressure (DBP) ≥ 85 mmHg and systolic blood pressure (SBP) ≥ 130 mmHg; having two or more of these was considered metabolically unhealthy. The cut-off value of WC for abdominal obesity was ≥ 80 cm for females and ≥ 85 cm for males [26]. All subjects were divided into four phenotypes based on the presence ( +) or absence ( − ) of metabolically healthy/unhealthy (MH) and abdominal obesity (AO) at baseline: metabolically healthy + non-abdominal obesity (MH^−^AO^−^); metabolically healthy + abdominal obesity (MH^−^AO^+^); metabolically unhealthy + non-abdominal obesity (MH^+^AO^−^); metabolically unhealthy + abdominal obesity (MH^+^AO^+^).

Drinking status was divided into no drinking habits or small (< 40 g/w) drinking, light (40–139 g/w) drinking, and moderate (140–209 g/w) drinking according to weekly alcohol consumption.

Smoking status was divided into never smoking, smoking cessation, and current smoking.

Exercise habits: Subjects who participated in any form of physical activity at least once a week were considered to have exercise habits.

### Diagnosis of NAFLD

Abdominal ultrasonography was performed on the subjects by professional imaging technicians, and experienced gastroenterologists scored and made a diagnosis based on the ultrasonographic images without knowing the purpose of the study and other clinical information about the subjects. The scoring criteria were (1) blurred blood vessels (0–1 points); (2) liver brightness (0–4 points); (3) deep attenuation (0–2 points); (4) liver and kidney echo contrast (0–4 points). If the total score of these four items was greater than 2, it was diagnosed as NAFLD [27].

### Statistical analysis

Considering that there are significant differences between males and females in terms of body composition, fat deposition patterns, and the impact of obesity on health [28], all analyses in this study were performed separately on males and females. Baseline information for all subjects was described by grouping according to the four abdominal obesity phenotypes described above. In this study, data for all subjects were analyzed using Empower (R) (version 2.0) and R (version 3.4.3) statistical software. The main analysis process was as follows:

Descriptive analysis: QQ plot and Kolmogorov–Smirnov test were used to assess the normality of continuous variables, and Kruskal Wallis H test or one-way ANOVA was used to compare between groups. Exercise habits, smoking, and drinking status were described as frequency (%) and differences between groups were compared using the Pearson χ2 test.

Correlation analysis: Univariate and multivariate logistic regression models were established to explore the correlation between different abdominal obesity phenotypes and NAFLD risk, and their corresponding odds ratio (OR) and 95% confidence interval (CI) were recorded. Before the establishment of the multivariate logistic regression models, we checked for collinearity between all covariates [29]; the covariates with a variance inflation factor (VIF) greater than 5 were considered collinear and were not included in the subsequent model. Three multivariate logistic regression models were established to evaluate the relationship of different abdominal obesity phenotypes with NAFLD [30]. In Model 1, we adjusted for exercise habits, age, height and BMI. Considering the influence of liver enzyme metabolism and blood glucose metabolism on NAFLD risk, model 2 further adjusted ALT, GGT, AST, FPG and HbA1c based on model 1. To further explore the independent association between abdominal obesity phenotypes and NAFLD risk, model 3 adjusted all non-collinear variables on the basis of model 2.

## Results

### Baseline characteristics

Of the 14,251 subjects, 7,411 (52%) were males (mean age 43.82) and 6,840 (48%) were females (mean age 43.22). As shown in Table 1, the prevalence of abdominal obesity in female subjects was 14.96%, and the prevalence of metabolically unhealthy status was 20.54%. Among females, 4,567 (66.77%) were classified as MH^−^AO^−^, 868 (12.69%) as MH^−^AO^+^, 1,250 (18.28%) as MH^+^AO^−^, and 155 (2.26%) as MH^+^AO^+^; the prevalence of NAFLD in these four phenotypes was 1.73%, 24.42%, 7.60%, 59.35%, respectively.

**Table 1:** Characteristics of study subjects based on abdominal obesity^a^ and metabolically healthy/unhealthy^b^ for females

Variables	Total (n = 6840)	Metabolically healthy		Metabolically unhealthy	
		MH^−^AO^−^ (n = 4567)	MH^−^AO^+^ (n = 868)	MH^+^AO^−^ (n = 1250)	MH^+^AO^+^ (n = 155)
Age, years	43.22 (8.78)	42.03 (8.40)	46.18 (8.56)	44.82 (9.23)	48.97 (9.00)
Height, cm	158.28 (5.38)	158.39 (5.36)	158.91 (5.43)	157.52 (5.43)	157.57 (4.81)
Weight, kg	52.65 (7.85)	50.19 (5.64)	63.45 (7.88)	52.30 (6.14)	67.20 (9.22)
BMI, kg/m2	21.01 (2.93)	20.00 (2.00)	25.13 (2.89)	21.08 (2.29)	27.06 (3.38)
WC, cm	71.67 (8.07)	68.72 (5.52)	85.05 (5.09)	71.13 (5.49)	88.14 (7.56)
ALT, U/L	14.00 (11.00–17.00)	13.00 (11.00–16.00)	15.00 (12.00–20.00)	13.00 (11.00–17.00)	19.00 (14.00–26.00)
AST, U/L	16.00 (13.00–19.00)	16.00 (13.00–19.00)	17.00 (14.00–20.00)	16.00 (13.00–19.00)	18.00 (15.00–22.00)
GGT, U/L	12.00 (10.00–15.00)	11.00 (9.00–14.00)	13.00 (11.00–18.00)	12.00 (10.00–15.00)	16.00 (13.00–22.00)
HDL-C, mmol/L	1.64 (0.38)	1.76 (0.33)	1.51 (0.33)	1.36 (0.38)	1.15 (0.20)
TC, mmol/L	5.09 (0.88)	5.03 (0.82)	5.33 (0.86)	5.04 (1.01)	5.69 (0.97)
TG, mmol/L	0.66 (0.41)	0.51 (0.37–0.69)	0.72 (0.52–1.02)	0.71 (0.50–1.04)	1.59 (0.97–2.02)
FPG, mmol/L	4.98 (0.39)	4.88 (0.33)	5.13 (0.38)	5.18 (0.45)	5.51 (0.36)
HbA1c, %	5.19 (0.32)	5.14 (0.30)	5.31 (0.32)	5.24 (0.34)	5.47 (0.34)
SBP, mmHg	109.28 (14.28)	105.81 (11.40)	117.16 (13.52)	113.73 (17.29)	131.27 (20.06)
DBP, mmHg	67.56 (9.75)	65.32 (8.03)	72.33 (9.21)	70.71 (11.69)	81.73 (12.79)
Exercise habits					
No	5761 (84.23%)	3812 (83.47%)	766 (88.25%)	1045 (83.60%)	138 (89.03%)
Yes	1079 (15.77%)	755 (16.53%)	102 (11.75%)	205 (16.40%)	17 (10.97%)
*Drinking status*					
Non or small	6451 (94.31%)	4295 (94.04%)	821 (94.59%)	1186 (94.88%)	149 (96.13%)
Light	389 (5.69%)	272 (5.96%)	47 (5.41%)	64 (5.12%)	6 (3.87%)
*Smoking status*					
Non	6036 (88.25%)	4048 (88.64%)	755 (86.98%)	1098 (87.84%)	135 (87.10%)
Past	406 (5.94%)	272 (5.96%)	61 (7.03%)	67 (5.36%)	6 (3.87%)
Current	398 (5.82%)	247 (5.41%)	52 (5.99%)	85 (6.80%)	14 (9.03%)
NAFLD	478 (6.99%)	79 (1.73%)	212 (24.42%)	95 (7.60%)	92 (59.35%)

Values were expressed as mean (standard deviation) or medians (quartile interval) or n (%).MH^−^AO^−^: metabolically healthy non-abdominal obese; MH^−^AO^+^: metabolically healthy abdominal obese; MH^+^AO^−^: metabolically unhealthy non-abdominal obese; MH^+^AO.^+^: metabolically unhealthy abdominal obese; BMI: body mass index; WC: waist circumference; ALT: alanine aminotransferase; AST: aspartate aminotransferase; GGT: gamma-glutamyl transferase; HDL-C: high-density lipoprotein cholesterol; TC: total cholesterol; TG: triglyceride; FPG: fasting plasma glucose; HbA1c: hemoglobin A1c; SBP: systolic blood pressure; DBP: diastolic blood pressure; NAFLD: non-alcoholic fatty liver disease

^a^Abdominal obesity was defined as waist circumference ≥ 80 cm

^b^Metabolically unhealthy was defined as having > one component (except waist circumference criteria) of metabolic syndrome according to the National Cholesterol Education Program Adult Treatment Panel III definition

Among males, the prevalence of abdominal obesity and metabolically unhealthy status was significantly higher (Table 2), 26.29% and 39.41%, respectively. The male subjects with MH^−^AO^−^, MH^−^AO^+^, MH^+^AO^−^, and MH^+^AO^+^ phenotypes accounted for 43.20%, 17.37%, 30.51%, and 8.92% of the total male population, respectively. Notably, regardless of the abdominal obesity phenotypes, the prevalence of NAFLD in males was higher than that in females with the same phenotype; among males, the prevalence of NAFLD was 9.93% in subjects with MH^−^AO^−^ phenotype, 50.54% in subjects with MH^−^AO^+^ phenotype, 25.49% in subjects with MH^+^AO^−^ phenotype, while the prevalence of NAFLD in subjects with MH^+^AO^+^ phenotype was as high as 73.22%. Moreover, in both sexes, compared with the MH^−^AO^−^ phenotype, the other three phenotypes tended to show higher WC, age, FPG, ALT, weight, TC, BMI, AST, TG, HbA1c, GGT, SBP, DBP levels, and higher NAFLD prevalence, while lower HDL-C levels and less physical exercise.

**Table 2:** Characteristics of study subjects based on abdominal obesity^c^ and metabolically healthy/unhealthy^b^ for males

Variables	Total (n = 7411)	Metabolically healthy		Metabolically unhealthy	
		MH^−^AO^−^ (n = 3202)	MH^−^AO^+^ (n = 1288)	MH^+^AO^−^ (n = 2260)	MH^+^AO^+^ (n = 661)
Age, years	43.82 (8.99)	42.34 (8.85)	44.72 (8.99)	45.05 (9.06)	45.01 (8.38)
Height, cm	170.81 (6.01)	170.75 (5.85)	172.42 (6.25)	169.71 (5.93)	171.75 (5.76)
Weight, kg	67.29 (9.98)	62.53 (6.96)	77.34 (8.78)	64.65 (6.76)	79.81 (9.42)
BMI, kg/m2	23.04 (3.00)	21.44 (2.07)	26.00 (2.50)	22.44 (2.01)	27.04 (2.75)
WC, cm	80.35 (7.94)	75.71 (5.28)	89.79 (4.75)	78.27 (4.69)	91.58 (5.74)
ALT, U/L	20.00 (15.00–28.00)	18.00 (14.00–23.00)	25.00 (19.00–35.00)	20.00 (16.00–27.00)	30.00 (22.00–45.00)
AST, U/L	18.00 (15.00–22.00)	17.00 (14.00–21.00)	20.00 (16.00–25.00)	18.00 (15.00–22.00)	21.00 (17.00–27.00)
GGT, U/L	19.00 (14.00–27.00)	16.00 (13.00–22.00)	22.00 (17.00–32.00)	19.00 (15.00–28.00)	28.00 (20.00–39.00)
HDL-C, mmol/L	1.29 (0.34)	1.45 (0.31)	1.22 (0.25)	1.20 (0.34)	1.00 (0.21)
TC, mmol/L	5.16 (0.86)	5.01 (0.81)	5.30 (0.84)	5.18 (0.89)	5.50 (0.89)
TG, mmol/L	1.10 (0.72)	0.71 (0.53–0.97)	1.05 (0.76–1.39)	1.10 (0.72–1.72)	1.89 (1.33–2.40)
FPG, mmol/L	5.30 (0.37)	5.12 (0.28)	5.32 (0.33)	5.46 (0.38)	5.58 (0.32)
HbA1c, %	5.17 (0.32)	5.10 (0.29)	5.21 (0.32)	5.20 (0.33)	5.30 (0.35)
SBP, mmHg	118.23 (13.99)	112.88 (10.69)	121.48 (12.43)	120.17 (14.89)	131.21 (15.55)
DBP, mmHg	74.41 (9.85)	70.51 (7.59)	76.70 (8.56)	75.89 (10.51)	83.72 (10.54)
Exercise habits					
No	6020 (81.23%)	2486 (77.64%)	1100 (85.40%)	1860 (82.30%)	574 (86.84%)
Yes	1391 (18.77%)	716 (22.36%)	188 (14.60%)	400 (17.70%)	87 (13.16%)
Drinking status					
Non or small	5354 (72.24%)	2324 (72.58%)	921 (71.51%)	1613 (71.37%)	496 (75.04%)
Light	1369 (18.47%)	606 (18.93%)	228 (17.70%)	426 (18.85%)	109 (16.49%)
Moderate	688 (9.28%)	272 (8.49%)	139 (10.79%)	221 (9.78%)	56 (8.47%)
Smoking status					
Non	2710 (36.57%)	1256 (39.23%)	443 (34.39%)	787 (34.82%)	224 (33.89%)
Past	2153 (29.05%)	891 (27.83%)	436 (33.85%)	641 (28.36%)	185 (27.99%)
Current	2548 (34.38%)	1055 (32.95%)	409 (31.75%)	832 (36.81%)	252 (38.12%)
NAFLD	2029 (27.38%)	318 (9.93%)	651 (50.54%)	576 (25.49%)	484 (73.22%)

Abbreviations as in Table 1

^c^Abdominal obesity was defined as waist circumference ≥ 85 cm

^b^Metabolically unhealthy was defined as having > one component (except waist circumference criteria) of metabolic syndrome according to the National Cholesterol Education Program Adult Treatment Panel III definition

### Univariate analysis of the risk of NAFLD

Table 3 shows the correlation of covariates with NAFLD risk in univariate logical regression analysis. We found that among the female subjects, except for exercise habits and smoking status, the other covariates were significantly correlated with NAFLD risk. The abdominal obesity phenotype was highly associated with an increased risk of NAFLD. Compared with the MH^−^AO^−^ phenotype, the risk of NAFLD in female subjects with the MH^+^AO^+^ phenotype increased by 81.96 times (OR 82.96, 95%CI 56.16, 122.55), those with the MH^+^AO^−^ phenotype increased by 3.67 times (OR 4.67, 95%CI 3.44, 6.34), and those with the MH^−^AO^+^ phenotype increased by 17.36 times (OR 18.36, 95%CI 14.00, 24.07). Also, it is worth noting that compared with the MH^−^AO^−^ phenotype, the association of abdominal obesity phenotypes with NAFLD risk was relatively attenuated in male subjects. The OR value and 95%CI for the MH^−^AO^+^, MH^+^AO^−^, and MH^+^AO^+^ phenotypes were 9.27 (7.90, 10.87), 3.10 (2.67, 3.60), 24.80 (20.15, 30.52), respectively.

**Table 3:** Univariate analysis of the association between NAFLD and baseline variables

	Female		Male	
	OR (95%CI)	*P*-value	OR (95%CI)	*P-*value
Age	1.06 (1.05, 1.07)	< 0.0001	1.00 (1.00, 1.01)	0.0848
Weight	1.16 (1.14, 1.17)	< 0.0001	1.12 (1.11, 1.13)	< 0.0001
Height	0.96 (0.94, 0.97)	< 0.0001	0.99 (0.98, 1.00)	0.0841
BMI	1.58 (1.53, 1.64)	< 0.0001	1.62 (1.58, 1.67)	< 0.0001
WC	1.19 (1.17, 1.21)	< 0.0001	1.19 (1.18, 1.20)	< 0.0001
*Abdominal obesity phenotypes*				
MH^−^AO^−^	Ref		Ref	
MH^−^AO^+^	18.36 (14.00, 24.07)	< 0.0001	9.27 (7.90, 10.87)	< 0.0001
MH^+^AO^−^	4.67 (3.44, 6.34)	< 0.0001	3.10 (2.67, 3.60)	< 0.0001
MH^+^AO^+^	82.96 (56.16, 122.55)	< 0.0001	24.80 (20.15, 30.52)	< 0.0001
ALT	1.07 (1.06, 1.09)	< 0.0001	1.09 (1.09, 1.10)	< 0.0001
AST	1.05 (1.03, 1.06)	< 0.0001	1.08 (1.07, 1.09)	< 0.0001
GGT	1.04 (1.03, 1.05)	< 0.0001	1.02 (1.02, 1.03)	< 0.0001
HDL-C	0.08 (0.06, 0.11)	< 0.0001	0.10 (0.08, 0.12)	< 0.0001
TC	1.82 (1.65, 2.01)	< 0.0001	1.62 (1.53, 1.73)	< 0.0001
TG	7.74 (6.37, 9.41)	< 0.0001	3.05 (2.80, 3.32)	< 0.0001
FPG	7.27 (5.71, 9.25)	< 0.0001	3.76 (3.24, 4.36)	< 0.0001
HbA1c	12.35 (9.06,16.83)	< 0.0001	4.22 (3.56, 4.99)	< 0.0001
*Exercise habits*				
NO	Ref		Ref	
Yes	0.88 (0.67, 1.14)	0.3357	0.71 (0.62, 0.82)	< 0.0001
*Drinking status*				
Non or small	Ref		Ref	
Light	0.45 (0.25, 0.78)	0.0047	0.57 (0.50, 0.66)	< 0.0001
Moderate	NA	NA	0.55 (0.45, 0.67)	< 0.0001
*Smoking status*				
Non	Ref		Ref	
Past	0.83 (0.54, 1.26)	0.3748	1.03 (0.91, 1.17)	0.6474
Current	0.96 (0.64, 1.43)	0.8266	0.89 (0.79, 1.01)	0.0691
SBP	1.05 (1.05, 1.06)	< 0.0001	1.04 (1.04, 1.05)	< 0.0001
DBP	1.08 (1.07, 1.09)	< 0.0001	1.06 (1.05, 1.07)	< 0.0001

Abbreviations as in Table 1; OR: Odds ratios; CI: confidence interval

### Association of abdominal obesity phenotypes with NAFLD

In the collinearity screening before building the multivariate logistic regression models (Additional file 1: Table S1), WC, body weight, and DBP were not included in subsequent models due to VIF greater than 5. In multivariate logistic regression models 1 and 2 (Table 4), all other phenotypes significantly increased the risk of NAFLD in both sexes compared with the MH^−^AO^−^ phenotype (All *P* < 0.001). Model 3 further adjusted for all non-collinear variables based on Model 2, and among female subjects, those with MH^+^AO^+^ and MH^−^AO^+^ phenotypes had a significantly higher risk of NAFLD than those with MH^−^AO^−^ phenotype [(OR 2.34, 95%CI 1.32, 4.17) and (OR 2.13, 95%CI 1.47, 3.09)]; similarly, in male subjects, the OR and 95%CI for the risk of NAFLD associated with MH^−^AO^+^ and MH^+^AO^+^ were 1.42 (1.13, 1.78), 1.47 (1.08, 2.01), respectively. However, the MH^+^AO^−^ phenotype was not associated with an increased risk of NAFLD in both sexes.

**Table 4:** Odds ratios for NAFLD events based on baseline abdominal obesity^a^ or abdominal obesity^c^ and metabolically healthy/unhealthy.^b^ phenotype in females and males

		Odds ratios (95% confidence interval)		
	Crude model	Model 1	Model 2	Model 3
*Female*				
MH^−^AO^−^	Ref	Ref	Ref	Ref
MH^−^AO^+^	18.36 (14.00, 24.07)^*^	3.01 (2.12, 4.29)^*^	2.48 (1.73, 3.57)^*^	2.13 (1.47, 3.09)^*^
MH^+^AO^−^	4.67 (3.44, 6.34)^*^	2.85 (2.08, 3.91)^*^	2.36 (1.68, 3.30)^*^	1.37 (0.93, 2.02)
MH^+^AO^+^	82.96 (56.16, 122.55)^*^	8.64 (5.34, 13.99)^*^	5.84 (3.48, 9.80)^*^	2.34 (1.32, 4.17)^*^
*Male*				
MH^−^AO^−^	Ref	Ref	Ref	Ref
MH^−^AO^+^	9.27 (7.90, 10.87)^*^	2.04 (1.66, 2.50)^*^	1.64 (1.31, 2.04)^*^	1.42 (1.13, 1.78)^*^
MH^+^AO^−^	3.10 (2.67, 3.60)^*^	2.26 (1.93, 2.64)^*^	1.82 (1.52, 2.18)^*^	1.19 (0.97, 1.45)
MH^+^AO^+^	24.80 (20.15, 30.52)^*^	4.34 (3.36, 5.59)^*^	2.79 (2.10, 3.71)^*^	1.47 (1.08, 2.01)^*^

Abbreviations as in Table 1;

Model 1 adjusted for age, height, exercise habits, and BMI;

Model 2 adjusted for model 1 plus ALT, AST, GGT, FPG, and HbA1c;

Model 3 adjusted for model 2 plus TC, TG, HDL-C, drinking status, and smoking status;

^*^Compared to the MH^−^AO^−^ phenotype, *P* < 0.05

## Discussion

In the present study, we conducted a cross-sectional analysis of the physical examination data of the general population and for the first time identified associations between abdominal obesity phenotypes and NAFLD. Our study found that in both sexes, except for the MH^+^AO^−^ phenotype, both the MH^−^AO^+^ phenotype and MH^+^AO^+^ phenotype were closely related to NAFLD risk. Furthermore, notably, this association appeared to be stronger in females.

NAFLD is the most common chronic liver disease in the world [1, 2], characterized by oxidative stress, inflammation, and fibrosis of hepatocytes, and is the main cause of end-stage liver disease, primary liver cancer, and liver transplantation [5, 31, 32]. Obesity and metabolic syndrome are important risk factors for NAFLD, and the coexistence of multiple diseases with metabolic syndrome and obesity as the main manifestation is the most typical clinical feature of NAFLD patients [9, 10, 33]. In recent years, obesity and metabolic syndrome are often widely studied as the phenotypes of other metabolic diseases; metabolic obesity phenotypes have been found in numerous observational studies to identify a variety of metabolic diseases and can be indicative of future risk [14, 15, 34–36]. However, increasing evidence suggested that there was a stronger association between central obesity and the health outcomes of the disease and that excessive visceral fat deposition rather than subcutaneous fat was an important risk factor associated with IR and metabolic abnormalities [19, 37]. WC, a useful anthropometric parameter for assessing visceral fat, has been widely used in metabolic disease research in recent years. Several cohort studies have identified a favorable correlation of the abdominal obesity phenotype with type 2 diabetes, all-cause mortality, and risk of cardiometabolic disease [17, 18, 38]. However, the relationship of NAFLD with abdominal obesity phenotypes remains unreported. Based on our current findings, we have identified for the first time the association of NAFLD with abdominal obesity phenotypes and found that even abdominal obesity patients with healthy metabolic status had a high risk of NAFLD.

MHO is one of the most widely discussed topics in recent years. Several studies have suggested that high BMI in the general population under metabolically healthy conditions usually did not increase the risk of metabolic diseases [12, 13, 39]; of course, some studies contained opinions to the contrary [15, 34]. Most notably, in a 30-year follow-up study by ArnlövJ et al., it was found that even in overweight and obese individuals without metabolic syndrome, the risk of cardiovascular disease was increased [15]. Further studies have shown that the MHO phenotype was not a static obesity phenotype; patients with the MHO phenotype typically lost metabolic health status with long-term follow-up. This phenomenon was more pronounced in patients with the MH^−^AO^+^ phenotype and appeared to be caused by visceral fat deposition [14, 16, 40]. In the current study, we specifically assessed the association between the MH^−^AO^+^ phenotype and NAFLD, and we found that subjects with the MH^−^AO^+^ phenotype had a higher risk of developing NAFLD than those with the MH^−^AO^−^ phenotype, and moreover, they tended to have older age, less physical activity, lower HDL-C levels, and higher levels of adverse metabolic parameters, although these parameters were mostly within normal ranges. Therefore, we speculated that it was these adverse metabolic characteristics that contributed to the difficulty of maintaining metabolic health status in patients with the MH^−^AO^+^ phenotype and put them at increased risk of NAFLD.

In the present study, all subjects, whether metabolically healthy or non-metabolically healthy, had an increased risk of NAFLD as long as abdominal obesity was present. Although the underlying mechanism of abdominal obesity phenotype leading to NAFLD remains unclear, WC may be the strongest mediator between IR and NAFLD [20, 41]. When abdominal obesity patients are in a state of IR, liver de novo lipogenesis is increased, the oxidative decomposition of free fatty acids is inhibited, and a large amount of adipose tissue is decomposed. The above pathological changes will produce many risk factors for liver metabolisms, such as increased intrahepatic lipogenesis, fat deposition, and increased intrahepatic free fatty acid levels; these responses lead to disturbances in hepatic lipid metabolism, and increase lipotoxicants, and ultimately cause NAFLD [6]. In the current study, we have observed associations of NAFLD risk with the abdominal obesity phenotype in both sexes, but from the results, the NAFLD risk associated with the abdominal obesity phenotype appeared to be higher in females, although the prevalence of NAFLD in males was almost as high as in females 4 times (27.38% vs 6.99%); this similar phenomenon has also been reported in some previous studies [42, 43]. Sex differences in NAFLD risk may involve multiple mechanisms, of which the sexual dimorphism exhibited by regional adiposity and the benefits of estrogen in hepatic fat metabolism may be important reasons. It is well known that females and males have significant differences in body fat distribution [28], with the female having a greater ability to store fat in the subcutaneous compartment, while males are more inclined to store fat in internal organs. For any given amount of fat, males typically have twice as much visceral fat as females [44]; while more visceral fat content generally means higher hepatic and peripheral IR [45], which may partially explain the higher prevalence of NAFLD in males than in females. In addition, recent experimental studies have shown that large amounts of estrogen in premenopausal women can promote β-oxidation of free fatty acids in the liver, regulate energy homeostasis in the body, inhibit adipose tissue breakdown, and enhance adipose tissue insulin sensitivity, reducing peripheral delivery of free fatty acids to the liver, thereby exerting an anti-hepatic steatosis effect [46]. Furthermore, a number of observational studies have also found that the prevalence of NAFLD in postmenopausal females was significantly increased, which indirectly confirmed the protective effect of endogenous estrogen on NAFLD [47, 48]. The decline of estrogen levels in females after menopause and the emergence of postmenopausal metabolic syndrome have resulted in an increased risk of NAFLD [49], therefore, the reproductive status may be an important factor affecting the risk of NAFLD in females. However, due to the lack of information on female reproductive status in the current study data, we were unable to consider the effect of female reproductive status on NAFLD risk in the association analysis, which may be an important reason for the higher risk of NAFLD associated with the abdominal obesity phenotypes in females than in males, as the female reproductive status may partially mediate the association between the female abdominal obesity phenotypes and NAFLD; the impact of female reproductive status on NAFLD risk needs to be further discussed in future studies. Additionally, considering that the susceptibility of both sexes to NAFLD and its risk factors is quite different at different ages, we continued to analyze the possible interaction between age and sex on the impact of NAFLD risk (Additional file 1: Table S2); The results showed that there was an interaction between age and sex (*P* for interaction = 0.0466), and the risk of NAFLD in females was significantly higher than that in males. The specific reasons for the stronger association of the abdominal obesity phenotype with NAFLD in females are unknown, but excessive obesity may play a more important role in the development of fatty liver disease in females; further studies are needed to explain the sex-related differences in NAFLD.

Although abdominal obesity appears to be a major risk factor in the association of abdominal obesity phenotypes with NAFLD risk, subjects with the MH^+^AO^+^ phenotype had a higher risk of NAFLD than those with the MH^−^AO^+^ phenotype (OR: female 2.34 vs 2.13; male 1.47 vs 1.42); and in both sexes, the prevalence of NAFLD in patients with the MH^+^AO^+^ phenotype was the highest of all phenotypes (male: 73.22%; female: 59.35%). Therefore, the combination of abdominal obesity and metabolic status into abdominal obesity phenotypes can identify more individuals with NAFLD risk. In addition, WC, the index defining abdominal obesity, and FPG, HDL-C, TG, SBP, DBP, the diagnostic index of metabolic syndrome, belong to routine physical examination, so the information on the abdominal obesity phenotype of each subject can be obtained without additional measurement cost, which provides a simple and practical tool for the prevention and screening of NAFLD in clinical practice and large-scale epidemiological studies. The use of abdominal obesity phenotypes may encourage clinicians to take early targeted interventions for individuals with the MH^−^AO^+^ and MH^+^AO^+^ phenotype, improve the awareness of disease prevention in patients with adverse abdominal obesity phenotypes, and reduce the economic cost of treating NAFLD and its complications.

## Limitation

Some limitations are worth noting: First, because this study is a cross-sectional design, we could only demonstrate an association between abdominal obesity phenotypes and NAFLD, but not a causal relationship between them; second, we did not measure subjects' insulin levels also did not measure levels of inflammatory-related factors, thus, this may lead to suboptimal identification of the metabolically healthy phenotype; third, this study is based on the results of an abdominal ultrasound to diagnose NAFLD, and liver biopsy was not performed to confirm the diagnosis. However, it is unethical to use invasive testing modalities in large populations, and abdominal ultrasonography, with its high sensitivity and specificity for the detection of fatty liver, has also been used in many population-based studies; fourth, although all known risk factors have been adjusted to the greatest extent possible, it was inevitable that some covariate information cannot be measured and obtained [50].

## Conclusion

All in all, the current study demonstrated that people with the MH^−^AO^+^ phenotype and MH^+^AO^+^ phenotype had a higher risk of NAFLD than people with the MH^−^AO^−^ phenotype, especially in females. These findings highlight the importance of abdominal obesity as a risk factor for NAFLD and call into question the concept of "metabolically healthy obesity". Therefore, the risk screening of NAFLD and related metabolic diseases in people with abdominal obesity should be strengthened, and the occurrence of adverse metabolic health outcomes should be prevented through active lifestyle interventions.

## Supplementary Information

**Additional file 1:** Supplementary Tables. **Supplementary Table 1**. Collinearity diagnostics steps. **Supplementary Table 2**. Stratified association between age and NAFLD by sex.

## Data Availability

The datasets that support the conclusions of this article can be found in the Dryad repository, and we confirm that this data set is publicly available in the Dryad database (https://datadryad.org/stash/dataset/doi:10.5061%2Fdryad.8q0p192).

## Abbreviations

BMI: Body mass index
MH^−^AO^−^: Metabolically healthy non-abdominal obesity
MH^−^AO^+^: Metabolically healthy abdominal obesity
MH^+^AO^−^: Metabolically unhealthy non-abdominal obesity
MH^+^AO^+^: Metabolically unhealthy abdominal obesity
WC: Waist circumference
ALT: Alanine aminotransferase
AST: Aspartate aminotransferase
GGT: Gamma-glutamyl transferase
HDL-C: High-density lipoprotein cholesterol
TC: Total cholesterol
TG: Triglyceride
FPG: Fasting plasma glucose
HbA1c: Hemoglobin A1c
SBP: Systolic blood pressure
DBP: Diastolic blood pressure
NAFLD: Non-alcoholic fatty liver disease
IR: Insulin resistance
VIF: Variance inflation factor
MHO: Metabolically healthy obesity
