# Supplementary material for: Abdominal obesity phenotypes are associated with the risk of developing non-alcoholic fatty liver disease: insights from the general population
Source: BMC Gastroenterol. 2022 Jun 25;22:311. doi: 10.1186/s12876-022-02393-9 (PMC9233393; doi:10.1186/s12876-022-02393-9)
Supplement: Supplementary file 1 — Additional file 1: Supplementary Tables. Supplementary Table 1. Collinearity diagnostics steps. Supplementary Table 2. Stratified association between age and NAFLD by sex. [file 12876_2022_2393_MOESM1_ESM.docx]

Supplementary Table 1: Collinearity diagnostics steps.

|  | Variance inflation factor | | | |
| --- | --- | --- | --- | --- |
|  | Step 1 | Step 2 | Step 3 | Step 4 |
| Phenotypes | 1.9 | 1.9 | 1.9 | 1.9 |
| Sex | 3.4 | 3.3 | 3.3 | 3.3 |
| Age | 1.4 | 1.4 | 1.3 | 1.3 |
| BMI | 95.9 | 5 | 1.7 | 1.7 |
| WC | 6 | 6 | NA | NA |
| ALT | 4.1 | 4.1 | 4.1 | 4.1 |
| AST | 3.3 | 3.3 | 3.3 | 3.3 |
| Weight | 168.7 | NA | NA | NA |
| Exercise | 1 | 1 | 1 | 1 |
| GGT | 1.5 | 1.5 | 1.5 | 1.5 |
| HDL | 2 | 2 | 2 | 2 |
| TC | 1.5 | 1.5 | 1.5 | 1.5 |
| TG | 1.8 | 1.8 | 1.8 | 1.8 |
| FPG | 1.7 | 1.7 | 1.7 | 1.7 |
| HbA1C | 1.2 | 1.2 | 1.2 | 1.2 |
| Drinking | 1.2 | 1.2 | 1.2 | 1.2 |
| Smoking | 1.4 | 1.4 | 1.4 | 1.4 |
| SBP | 5.5 | 5.5 | 5.5 | 1.5 |
| DBP | 5.6 | 5.6 | 5.6 | NA |
| Height | 52 | 2.9 | 2.4 | 2.4 |

Note-1: Variance inflation factor = 1/(1-R^2^). Abbreviations as in Table 1.

Note-2: The variables with Variance inflation factor >5 will be regarded as collinear variables and cannot be included in the multiple regression model.

Supplementary Table 2: Stratified association between age and NAFLD by sex.

| Subgroup | No. of cases | unadjusted OR (95%CI) | adjusted OR (95%CI) | *P-*interaction |
| --- | --- | --- | --- | --- |
| Sex |  |  |  | 0.0466 |
| Male | 2029 (27.38%) | 1.00 (1.00, 1.01) | 1.02 (1.01, 1.03) |  |
| Female | 478 (6.99%) | 1.06 (1.05, 1.07) | 1.03 (1.02, 1.05) |  |

OR: Odds ratios; CI: confidence interval.

Note: Model adjusted for the same covariates as in model 3 (Table 4).
